# Layered Nano-Sheets: Synthesis and Applications

**DOI:** 10.3390/nano10030514

**Published:** 2020-03-12

**Authors:** Muralidharan Paramsothy

**Affiliations:** NanoWorld Innovations (NWI), 1 Jalan Mawar, Singapore 368931, Singapore; mpsothy@yahoo.co.uk

Extraordinary physical and chemical properties are enabled by two dimensional (2D) anisotropy and confinement effects in layered nano-sheet materials. Layered nano-sheet materials are 2D crystals possessing properties useful in applications ranging from electronics and energy storage to structural loads bearing nanocomposites. Thematically, six representative articles [1,2,3,4,5,6] have been published in this Special Issue:

Gao et al. [1] reported that low-temperature nitrogen plasma processing was conducted to modify graphitic carbon nitride (g-C_3_N_4_) to enhance electro-catalytic performance for the ***hydrogen evolution*** reaction (HER). The plasma incorporated nitrogen atoms onto the surface of g-C_3_N_4_. The modification improved surface hydrophilicity, electro-catalytic HER activity, and overall stability in HER after 2000 cycles. In the work by Zhao et al. [2], various ions including H_3_O^+^, Fe^3+^, Au^3+^, and Zn^2+^ were successfully encapsulated in graphene oxide nanoscrolls (GONSs). As the pH of the graphene oxide (GO) solution decreased from 5 to 0.15, the height of GONS was increased from ~50 to ~190 nm due to increased interlayer distance by encapsulating H_3_O^+^. GONSs with encapsulated metal ions were prepared by dissolving metal ions into a GO solution with a pH of 0.3. As-encapsulated metal ions were converted to metal or metal oxide nanoparticles when they were annealed at 480 °C for 30 min. By reducing GO to rGO with L-ascorbic acid in solution, H_3_O^+^ embedded rGONS were fabricated by molecular combing rGO solution with a pH of 0.3. The resistance-type ***pressure sensor*** based on the (rGONS)_0.3_ mesh showed good response to applied pressure on it compared to that of the sensor based on (rGONS)_5_ mesh without H_3_O^+^.

As reported by Gill-Castell et al. [3], functionalized nanofibrous membranes based on poly(vinyl alcohol) (PVA) or sulfonated poly(vinyl alcohol) (SPVA) combined with graphene oxide (GO) were developed by means of electrospinning. The subsequent crosslinking reaction with sulfosuccinic acid (SSA) brought thermal and hydrothermal stability as well as the required electrical insulator performance and proton conductivity to the nanofibers. The crosslinked nanofibrous membranes were stable during simulated service conditions. Although fiber diameter increased after immersion, the presence of GO and the use of SPVA contributed to higher diameter stability. The developed nanofibrous composite membranes were considered for ionic ***multi-layered polyelectrolyte*** application. Saeed et al. [4] reported that graphene nanoplatelets were functionalized following an oxidation–reduction process, and then dispersed in glycol as the base fluid. The characterization of the thermo-physical properties of these nanofluids was performed by determining their viscosity and thermal conductivity due to the relevant impact on practical applications related with ***fluid flow and heat transfer***. The effects of temperature and shearing time on viscosity were analyzed. Shear-thinning behaviour strongly influenced by temperature was exhibited.

In the work by Burguete et al. [5] for ***open technological*** application, a simple and reproducible method for the direct preparation of high purity silica-based micro/nanocharges was designed. The “modified atrane route” enabled a wide family of layered mesostructured silica derivatives to be obtained. Chemical homogeneity and good dispersion of the inorganic/organic functional groups was observed in the surfactant-assisted mesostructure formation. The main advantage of the resulting neutral-amine templated matrices (with regard to other natural or synthetic lamellar solids) was the ease of manufacture, modification, expansion, and adaptation to polymers, relevant to single one-pot procedures. 

Lv et al.’s [6] work involved graphene oxide (GO) nanosheets prepared using the Hummers method, which were found to easily aggregate in aqueous and cement composites. Using carboxymethyl chitosan (CCS) as a dispersant, few-layered GO nanosheets (1–2 layers) were obtained by forming CCS/GO intercalation composites. The testing results indicated that the few-layered GO nanosheets could uniformly spread in both aqueous and cement composites. A special feature was determined, namely that the microstructures consisted of regular-shaped crystals created by self-crosslinking. X-ray diffraction (XRD) results indicated that there was a higher number of cement hydration crystals in the GO/cement composites. It was also observed that partially-amorphous calcium-silicate-hydrate (C-S-H) gel turned into monoclinic crystals. Durability parameters such as penetration, freeze–thaw, carbonation, drying-shrinkage value, and pore structure showed marked improvement. The results indicated that it is possible to obtain cement composites with a compact microstructure and high ***structural load bearing*** performance by introducing CCS/GO intercalation composites.

**Note: *Bold italics*** highlight contemporary technological applications. 
